# Case Report: Multimodality imaging of a congenital ventricular septal aneurysm complicated by biventricular myocardial non-compaction in a child

**DOI:** 10.3389/fped.2025.1559102

**Published:** 2025-10-01

**Authors:** Jie Zhou, Jiao Chen

**Affiliations:** ^1^ Department of Ultrasonic Medicine, West China Second University Hospital of Sichuan University, Chengdu, China; ^2^ Key Laboratory of Birth Defects and Related Diseases of Women and Children (Sichuan University), Ministry of Education, Chengdu, China; ^3^ Tibet Autonomous Region Women’s and Children’s Hospital, West China Second University Hospital of Sichuan University, Lhasa, China; ^4^ Xizang Region Child Development Clinical Medical Research Center, Xizang, China

**Keywords:** congenital ventricular aneurysm, left ventricle, multimodality imaging, biventricular myocardial non-compaction, child

## Abstract

Congenital ventricular aneurysm is a rare malformation affecting all cardiac chambers, and is most frequently observed in the left ventricle. Congenital ventricular aneurysm is characterized by localized bulges in the ventricular wall, typically distinct from the normal myocardium, and it shows akinesia or dyskinesia. Using multimodality imaging, we diagnosed a case of ventricular septal aneurysm complicated by biventricular myocardial non-compaction in a child. After excluding various potential causes, we considered a congenital developmental abnormality as the most likely pathogenic factor.

## 1 Introduction

A left ventricular (LV) aneurysm typically occurs in middle-aged and elderly patients with coronary heart disease. It is characterized by ventricular expansion in the infarcted region, accompanied by myocardial thinning, necrosis, and subsequent replacement with fibrous tissue (1). The thinning of the ventricular wall in affected areas leads to impaired or paradoxical heart contraction (1). This condition most frequently arises as a complication of acute myocardial infarction, though multiple other etiological factors have been identified. These include traumatic injury, hypertrophic cardiomyopathy, infectious processes such as tuberculosis and endocarditis, inflammatory manifestations of connective tissue disorders, coronary vascular pathologies (notably Kawasaki disease), and congenital coronary artery origin anomalies (1). In contrast to these acquired forms, congenital ventricular aneurysm represents an exceptionally rare clinical entity, with an estimated prevalence of approximately 5/1,000,000 individuals, showing no sex predilection, and its etiology remains unclear (2).

Ventricular non-compaction cardiomyopathy is characterized by an abundance of prominent ventricular trabeculae and deep intertrabecular recesses. The pathogenesis of this condition is thought to be associated with disturbances in endocardial morphogenesis, leading to the stagnation of normal cardiac development (3).

We reported a case of LV congenital septal aneurysm complicated by biventricular non-compaction, as shown by multimodal imaging, in a boy. To the best of our knowledge, this finding has not been previously been reported.

## 2 Case description

**Figure 1:**
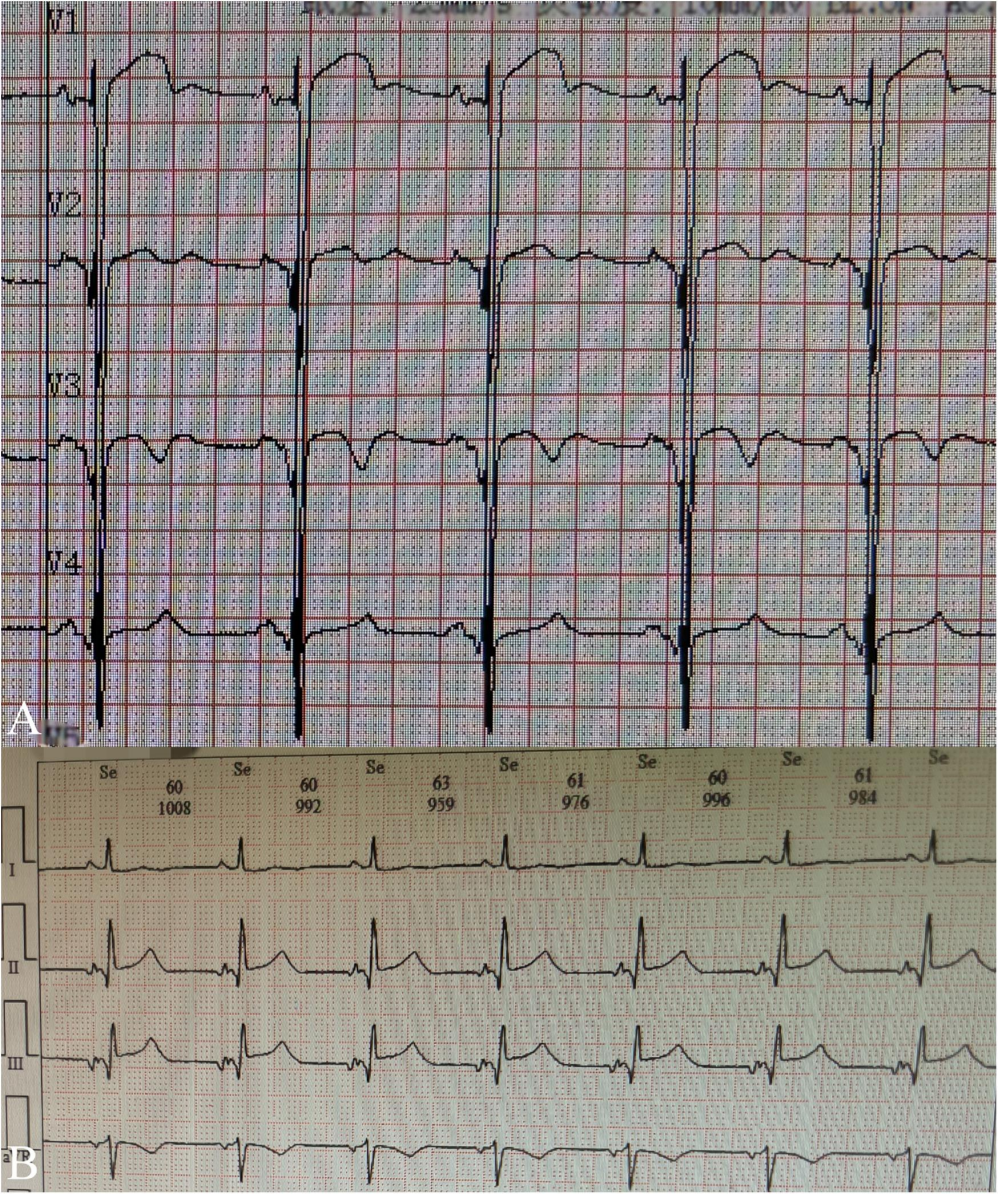
Electrocardiogram fingdings during hospitalization: **(A)** routine electrocardiogram shows abnormal Q-waves, with visible Q-waves in leads V2–V4 and ST segment elevation of 0.1–0.2 mV in leads V1–V4; **(B)** Excerpt from holter electrocardiogram monitoring reveals accelerated atrial ectopic rhythm.

**Figure 2:**
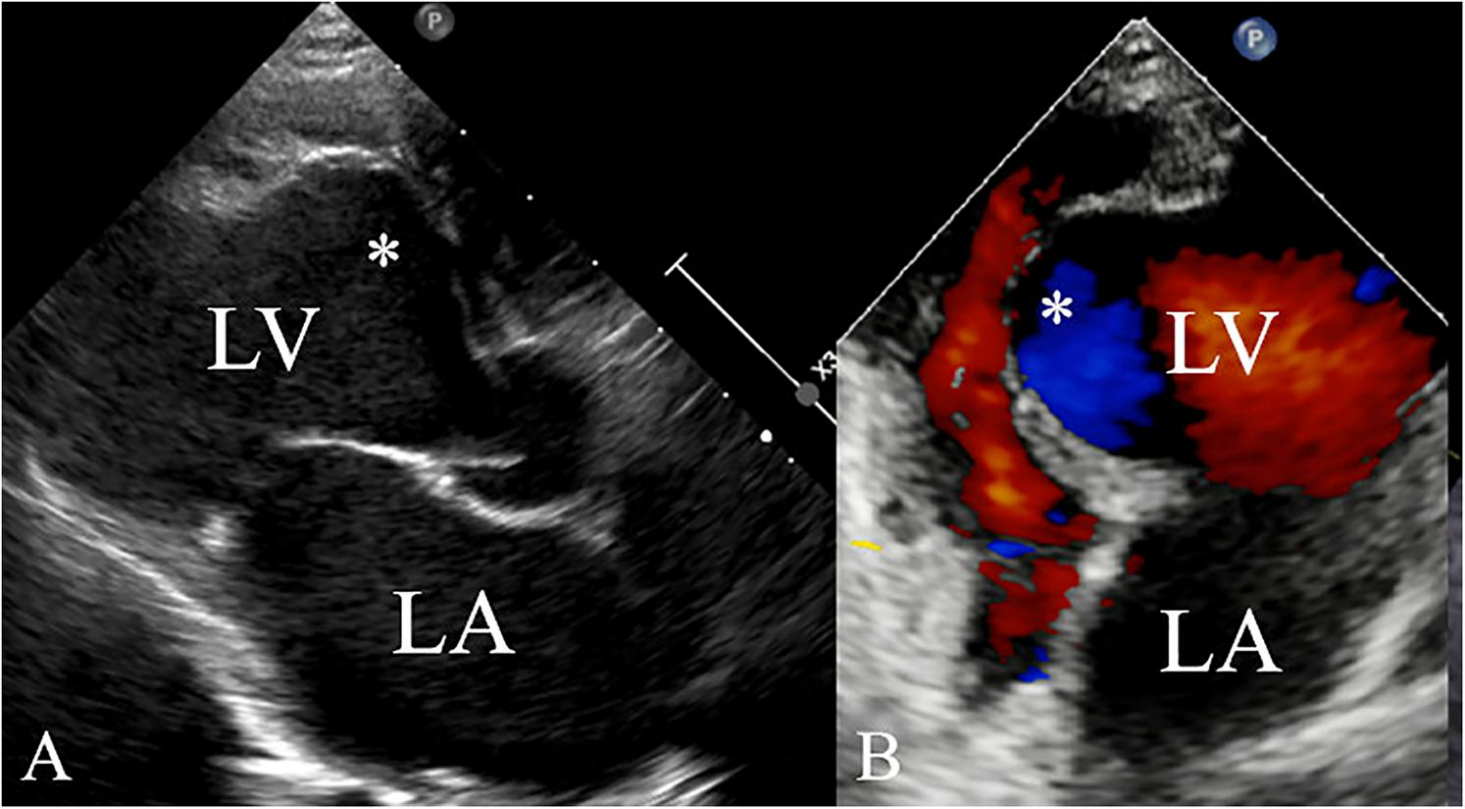
Ventricular aneurysm (*) detected by echocardiography **(A)** of the left ventricle in the long-axis view and **(B)** the apical four-chamber view.

**Figure 3:**
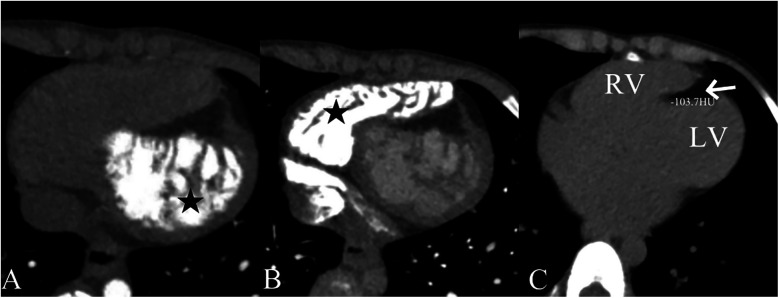
Computed tomographic angiography findings showing left **(A)** and right **(B)** ventricular myocardial non-compaction (★) and fat density shadows within the ventricular septal myocardium (arrow). The computed tomographic value was −103.7 HU **(C)**.

**Figure 4:**
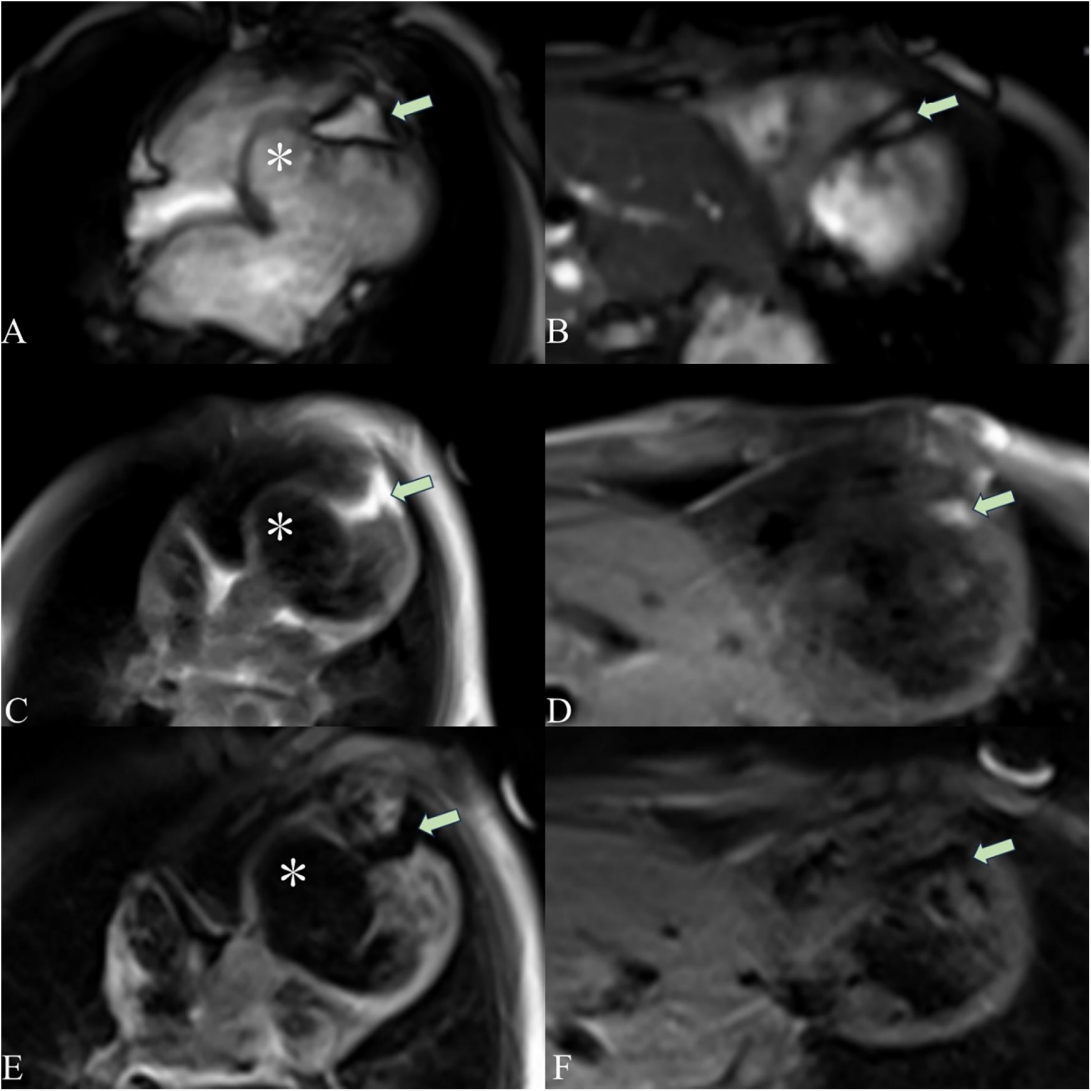
Cardiac magnetic resonance imaging showing a ventricular aneurysm (*) and fatty infiltration. The cardiac magnetic resonance four-chamber view cine sequence **(A)**, T1-weighted image **(C)**, and T2 fat-suppression sequence **(E)** show a ventricular aneurysm (*) and fatty signals (arrows) in the middle and apical regions of the LV septum. The short-axis cine sequence **(B)**, T1-weighted image **(D)**, and T2 fat-suppression sequence **(F)** demonstrate fatty signals (arrows) in the middle and apical regions of the LV septum. On T1-weighted imaging, fat appears as a high-signal intensity area. The T2 fat-suppression sequence suppresses fat signals.

A 5-year-old boy presented with a history of cough and two fever episodes within 3 weeks. Upon admission, his vital signs were stable (T: 36.5 ℃, HR 98 bpm, RR 20 bpm). A physical examination showed the absence of wheezing, with notable cardiothoracic findings. These findings included precordial prominence, cardiomegaly on percussion (documented as 0.5-cm lateral displacement of the apical impulse from the left midclavicular line at the 5th intercostal space), and a regular cardiac rhythm without audible murmurs. His personal history (including prenatal examinations) and familial medical histories were noncontributory. A chest x-ray showed an enlarged cardiac shadow and pneumonia. Electrocardiographic findings included abnormal Q-waves, with visible Q-waves in leads II, III, aVF, and V2–V4. Additionally, ST segment changes were observed (ST segment elevation of 0.1–0.2 mV in leads V1–V4) (Figure 1A), along with QTc prolongation, left atrial heterogeneity, and double-compartment hypertrophy. Echocardiography indicated left cardiac enlargement [Left atrial volume = 28.3 ml (*Z* score = 4.0), LVend-diastolic diameter = 42 mm (*Z* score = 5.6)], a ventricular septal aneurysm in the basal and intermediate segments measuring 30 mm × 13.8 mm × 19.0 mm (Figure 2), and biventricular myocardial non-compaction with decreased LV dysfunction [ejection fraction (EF) = 40%, average E/e' = 15.4]. Computed tomographic angiography was systematically conducted for an accurate morphological assessment of the ventricular aneurysm (including topographic localization, volumetric measurement, and perianeurysmal anatomy). This technique showed left cardiac enlargement, increased trabeculation in both ventricles with a grid-like pattern, and fat density shadows within the ventricular septal myocardium (Figure 3), and a ventricular septal aneurysm. A Holter electrocardiogram showed an accelerated atrial escape rhythm (Figure 1B). Magnetic resonance imaging showed left cardiac enlargement [Left atrial volume = 34.82 ml (*Z* score = 4.9), LV end-diastolic volume = 76.41 ml (*Z* score = 4.1)], local aneurysm formation in the ventricular septum, biventricular myocardial non-compaction, and fatty infiltration in the middle and apical regions of the LV septum (Figure 4). LV systolic function was impaired (LV ejection fraction = 41.1%) and the right ventricular ejection fraction was normal (55.4%). First-pass perfusion imaging showed a perfusion defect in the ventricular septum of the mid-left ventricle and apex. Delayed enhancement imaging indicated myocardial fibrosis in the above area, which was likely secondary to myocardial ischemia.

The patient showed an elevated brain natriuretic peptide concentration of 220.2 pg/ml. Coronary computed tomographic angiography was conducted because of the boy's myocardial ischemia, despite an echocardiogram indicating a normal coronary origin and proximal segment. This imaging technique showed a dominant left coronary artery and a thin right coronary artery. Subsequently, coronary angiography was carried out to exclude coronary artery disease. The angiography examination showed no filling defects in the left or right coronary arteries, with a thrombolysis in myocardial infarction blood flow classification of grade 3. Comprehensive evaluations, including a blood routine test, blood gas analysis, biochemical analysis, coagulation function tests, myocardial enzyme analysis, muscle enzyme analysis, rheumatism screening, full blood count, immune transfusion analysis, electrolytes, autoantibody testing, anti-neutrophil cytoplasmic antibody testing, genetic metabolic disease screening, a T-spot test, and a purified protein derivative test, showed no major abnormalities. These findings ruled out endocarditis, tuberculosis, connective tissue disease, and other inflammatory causes. Whole-exome sequencing of the proband identified multiple phenotype-associated genetic variants, with subsequent parental segregation analysis by Sanger sequencing. However, a comprehensive pathogenicity assessment in accordance with the ACMG/AMP guidelines showed that the classification of these variants was variants of uncertain significance. Notably, a comprehensive genomic analysis showed no detectable rare pathogenic variants associated with the boy's clinical presentation. Consequently, the cardiac condition in this case was attributed to congenital factors. The parents opted for conservative management, and based on the patient's examination outcomes and evolving condition, the medication regimen was sequentially adjusted from milrinone, furosemide, sulperazon, and sacubitril/valsartan to sacubitril/valsartan, spironolactone, hydrochlorothiazide, clopidogrel, digoxin, enalapril, and levocarnitine. This treatment resulted in a stable current condition with an improved LVEF (55%) at discharge. The patient has been followed up for one and a half years, continues to receive conservative treatment, and remains in stable condition (Table 1).

**Table 1:** Diagnosis and treatment course.

Time	Examinations	Treatment
Admission	Biochemical examinations: no major abnormalities in a routine blood test, blood gas analysis, biochemical analysis, coagulation function tests, and myocardial enzyme analysis, and an elevated brain natriuretic peptide concentration of 220.2 pg/ml.	Milrinone, furosemide, sacubitril/valsartan, and sulperazon were administered.
	Chest x-ray: enlarged cardiac shadow and pneumonia.	
	Electrocardiogram: abnormal Q-waves, with visible Q-waves and ST segment changes.	
	Echocardiography: left cardiac enlargement, a ventricular septal aneurysm and biventricular myocardial non-compaction with decreased LV dysfunction (LVEF = 40%, average E/e = 15.4).	
1 day from admission	Computed tomographic angiography: left cardiac enlargement, increased trabeculation in both ventricles with a grid-like pattern, and fat density shadows within the ventricular septal myocardium.	Furosemide infusion was discontinued and switched to oral hydrochlorothiazide/spironolactone, the milrinone dosage was reduced, and the other medications continued to be used.
	Magnetic resonance imaging: left cardiac enlargement, ventricular aneurysm formation in the septum, biventricular myocardial non-compaction and fatty infiltration in the middle and apical regions of the LV septum, impaired LV systolic function (LVEF = 41.1%), a perfusion defect, and myocardial fibrosis in the ventricular septum of the mid-left ventricle and apex.	
3 days from admission	Biochemical examinations: no major abnormalities in a muscle enzyme analysis, rheumatism screening, full blood count, and immune transfusion analysis.	The medications continued to be used.
5 days from admission	Biochemical examinations: no major abnormalities in electrolytes, autoantibody testing, and anti-neutrophil cytoplasmic antibody testing.	Milrinone was discontinued and the other medications continued to be used.
	T-spot test and PPD test: normal.	
	Holter electrocardiogram: accelerated atrial escape rhythm.	
	Echocardiography: LVEF = 47%.	
6 days from admission	Genetic metabolic disease screening: normal.	The medications continued to be used.
	Coronary CTA: a dominant left coronary artery and a thin right coronary artery.	
7 days from admission	Coronary angiography: no filling defects in the left or right coronary arteries, with a TIMI blood flow classification of grade 3.	Digoxin, enalapril, and levocarnitine were administered, with continued use of the other medications.
9 days from admission/discharge	Echocardiography: LVEF = 55%.	Discharge medications: sacubitril/valsartan, spironolactone, hydrochlorothiazide, clopidogrel, digoxin, enalapril, and levocarnitine.
1 year after discharge	Echocardiography: LVEF = 54%.	Digoxin, coenzyme Q10, levocarnitine oral solution, furosemide spironolactone, acepril (note: brand name for Perindopril, commonly used in clinical settings in China), potassium Chloride Extended-Release Tablets, clopidogrel Bisulfate Tablets
	Electrocardiogram: sinus rhythm with QS waves in leads V1–3 and abnormal Q waves in leads III, aVF, and V4.	
1.5 year after discharge	Echocardiography: LVEF = 55%.	The medications continued to be used.

LV, left ventricle; LVEF, left ventricular ejection fraction; CTA, computed tomographic angiography; PPD, purified protein derivative; TIMI, thrombolysis in myocardial infarction.

## 3 Discussion

Congenital ventricular aneurysm is rare and hypothesized to be associated with prenatal viral infections, coronary artery embolism due to placental sinus abnormalities or stenosis, and focal ventricular wall weakening resulting from intrinsic embryonic developmental anomalies (4). There is limited literature on ventricular aneurysms in children. Some reports have indicated that these aneurysms are predominantly located in the apex rather than the septum (5). Non-compaction of the myocardium is characterized by excessive trabeculation and predominantly involves the left ventricle. In some cases, the right ventricle may also be affected—either concurrently with the left ventricle or as an isolated manifestation. This condition can occur in isolation or coexist with other cardiomyopathies. With advances in imaging techniques, its detected incidence in the pediatric population has been increasing. The etiology is heterogeneous, encompassing both genetic and acquired forms (6, 7).

Only four cases (8–11) of ventricular aneurysm coexisting with myocardial non-compaction have been reported, with three cases in adults and one case in a child (11). The pediatric case involved a newborn with a congenital apex LV aneurysm and LV non-compaction, who presented with treatment-resistant heart failure and passed away at 5 months of age. Notably, ventricular septal aneurysms complicated by biventricular myocardial non-compaction have not previously been reported. In this case, the young patient presented with no notable personal or familial medical history. Following a chest x-ray indicating an enlarged cardiac shadow, subsequent examinations showed the presence of ventricular septal aneurysms complicated by biventricular non-compaction. Despite evidence of myocardial infarction and decreased cardiac function upon imaging assessment, the child remained asymptomatic. Coronary artery lesions and genetic abnormalities were ruled out, with no prior history of myocarditis or related trauma. Computed tomography and magnetic resonance imaging indicated fatty infiltration in the midventricular septum and apex. Consequently, we speculate that these concurrent lesions were due to a congenital developmental abnormality. We also speculate that the process of pathogenesis is relatively slow myocardial development of the corresponding segment of the ventricular septum owing to fatty infiltration of the ventricular septum, while the muscular ventricular septal segment continues to grow, resulting in local aneurysmal bulging of the ventricular septum to the right ventricle (12). Myocardial ischemia ensues because of damage to the microcirculation of the nondense and dense layers (13), as well as disruption of the cardiac spiral structure by the ventricular aneurysm over time (14). Consequently, this cascade of events may perpetuate a vicious cycle, ultimately culminating in myocardial infarction and scar formation. However, a limitation of this hypothesis is that it has not been confirmed by histopathology. We recommend that the patient be followed up regularly for a long time because he may be at risk for progressive cardiac deterioration and even death.

## 4 Conclusion

We report a case of LV septal aneurysm complicated by biventricular non-compaction, as shown by multimodal imaging, in a child. To the best of our knowledge, this condition has not previously been reported. Our case illustrates the feasibility of conservative treatment for such pediatric patients, though long-term follow-up remains necessary.

## Data Availability

The original contributions presented in the study are included in the article/Supplementary Material, further inquiries can be directed to the corresponding author.
